# Role of Scoring Systems in Prognosticating Outcomes of Patients With Acute Pancreatitis: A Prospective Cohort Study

**DOI:** 10.7759/cureus.79738

**Published:** 2025-02-27

**Authors:** Faiz Khan Yusufi, Atia Zaka-ur-Rab, Sheelu Shafiq Siddiqi, Khaliqur Rahmaan Siddiqui, Abhinava Kolari, Hamza Khan Yusufi

**Affiliations:** 1 General Surgery, Jawaharlal Nehru Medical College, Aligarh, IND; 2 Surgery, Jawaharlal Nehru Medical College, Aligarh, IND; 3 Rajiv Gandhi Centre for Diabetes and Endocrinology, Jawaharlal Nehru Medical College, Aligarh, IND

**Keywords:** acute pancreatitis, apache ii, bisap, ctsi, ranson score

## Abstract

Introduction: Acute pancreatitis (AP) is a common cause of emergency hospital admissions, putting a substantial burden on the healthcare system. The clinical course of AP is usually mild and often resolves without a sequel. Severe AP (SAP) is associated with an intense inflammatory response leading to localized or systemic complications and significant morbidity and mortality (American Gastroenterological Association). Early diagnosis and precise assessment of disease severity are imperative during initial evaluation in patients with AP, as it has a bearing on deciding the course of management and prognosticating the disease outcome.

Materials and methods: Eighty-six cases of AP treated in our institution between July 2022 and August 2024 were prospectively enrolled in the study. The patients underwent detailed clinical evaluation, and the Acute Physiology and Chronic Health Evaluation II (APACHE II), Bedside Index of Severity in Acute Pancreatitis (BISAP), and Ranson scores were calculated. Ranson was again calculated after 48 hours of admission. Contrast-enhanced computed tomography of the abdomen was done in all patients after 72 hours of the onset of pain, and a modified computed tomography severity index (mCTSI) was calculated. Data regarding patients was collected and correlated with the outcome.

Results: During the course of the disease, seven (8.1%) patients died, while 79 (91.9%) improved. The majority of the patients, five of the seven patients (71.4%) who died, had SAP. On ROC curve analysis, Ranson was found to be the best predictor of SAP (area under the curve (AUC): 0.97), followed by APACHE II (AUC: 0.95), mCTSI (AUC: 0.95), and BISAP (AUC: 0.87). mCTSI was found to be the best predictor of pancreatic necrosis (AUC: 0.94), followed by Ranson (AUC: 0.87), APACHE-II (AUC: 0.78), and BISAP (AUC: 0.52). APACHE II had a slight edge over the rest of the scoring system in mortality predicting (APACHE II AUC: 0.72 95% CI (0.58-0.85), BISAP AUC: 0.67 95% CI (0.52-0.80), Ranson AUC: 0.68 95% CI (0.54-0.80), and mCTSI AUC: 0.72 95% CI (0.58-0.85)).

Conclusion: The ROC curve analysis demonstrated that Ranson was superior to the other scoring systems for predicting severity, and APACHE II had the highest accuracy for mortality.

## Introduction

Acute pancreatitis (AP) is a common cause of emergency hospital admissions, putting a substantial burden on the healthcare system. Globally, the reported incidence of AP is around five to 35 cases per 100,000 people [1-4]. It has been predicted to increase in the coming years [2,3,5,6]. The disease severity ranges from mild to severe. The clinical course of AP is usually mild and often resolves without a sequel. Approximately 10-20% of the patients develop severe AP (SAP), in which there is an intense inflammatory response leading to localized or systemic complications and significant morbidity and mortality (American Gastroenterological Association) [5,7,8]. Despite advancements in medical treatment, patients with severe disease are still at risk of developing pancreatic necrosis, systemic inflammatory response syndrome, multi-organ failure, and a significantly high risk of mortality [1]. The reported incidence of severe AP is 12% in patients with sterile necrosis, 30% in infected necrosis, and 47% in patients with multi-organ failure [9]. It has been observed that patients with severe disease benefit substantially from the requisite prompt aggressive treatment in the intensive care unit [10]. Early diagnosis and precise assessment of disease severity are, therefore, imperative at the time of initial evaluation in patients with AP, as it has a bearing on deciding the course of management and prognosticating the disease outcome [11,12]. Since clinical judgment alone has not been found reliable enough to assess the severity of AP, several scoring systems, such as the Acute Physiology and Chronic Health Evaluation II (APACHE) II, Ranson, Bedside Index of Severity in Acute Pancreatitis (BISAP), and modified computed tomography severity index (mCTSI), have, therefore, been developed to evaluate the severity of disease to triage the patient of AP at the time of admission for optimizing the utilization of healthcare facilities. Few studies have compared the role of these scoring systems in prognosticating the disease severity of AP based on the Revised Atlanta Guidelines (2012) classification.

The present study was, therefore, undertaken to compare the accuracy of APACHE II, BISAP, Ranson, and mCTSI in predicting the severity of AP based on the Revised Atlanta Guidelines (2012) definitions.

## Materials and methods

After obtaining clearance from the Institutional Ethics Committee Jawaharlal Nehru Medical College and Hospital (approval number: IECJNMCH/848) and well-informed consent from patients, 86 cases of AP treated in our institution between July 2022 and August 2024 were prospectively enrolled. The patients underwent detailed clinical evaluation and were classified into having mild to moderate SAP and SAP as per the Revised Atlanta Guidelines (2012). The scores for APACHE II, BISAP, and Ranson were calculated. Ranson was again calculated after 48 hours of admission as per the recommendation of the AGA. Contrast-enhanced computed tomography of the abdomen was done in all patients after 72 hours of the onset of pain, and mCTSI was calculated. The outcome was assessed based on the length of hospital stay, in-hospital complications, and mortality.

Table 1 summarizes the diagnostic criteria, severity classifications, and complications of AP based on the Revised Atlanta Guidelines (2012) and various scoring systems, including Ranson, APACHE II, BISAP, and mCTSI.

**Table 1:** Definitions and criteria for AP severity and complications AP: acute pancreatitis, SAP: severe acute pancreatitis, CT: computed tomography, MRI: magnetic resonance imaging, APACHE II: Acute Physiology and Chronic Health Evaluation II, BISAP: Bedside Index of Severity in Acute Pancreatitis, mCTSI: Modified Computed Tomography Severity Index

Term	Definition
Diagnosis of AP	Confirmed when two of three criteria are present: (a) abdominal pain characteristic of AP, (b) serum amylase or lipase levels >3 times the upper limit of normal, and (c) characteristic findings of AP on abdominal ultrasonography, CT scan, or MRI [13]
Organ failure	Diagnosed based on the modified Marshal scoring system [13]
Local complications	Necrosis, abscess, and pseudocyst [13]
Systemic complications	Disseminated intravascular coagulation (platelet counts ≤ 100,000), fibrinogen <1 g/L, fibrin degradation products >80 µg/dL, and metabolic disturbances (calcium level ≤ 7.5 mg/dL) [13]
SAP	Persistent organ failure (lasting more than 48 hours) of a single or multiple organ failure [13]
Mild AP	Patients with AP have no local/systemic complications or organ dysfunction [13]
Moderately severe AP	Patients with AP having transient (lasting <48 hours) organ dysfunction or local/systemic complications [13]
Severe Ranson score	Patients with a Ranson score ≥3 assessed on admission and after 48 hours [14]
Severe APACHE II score	Patients having APACHE II ≥8 on admission [15,16]
Severe BISAP score	BISAP score ≥2 on admission [17]
Severe mCTSI	mCTSI score of 8-10, assessed after 72 hours of symptom onset [18,19]

Assessment of outcome

The following parameters for each episode of AP were collected: length of hospital stay, in-hospital, presence of organ failure, and local complications such as peripancreatic fluid collections, abscess, necrosis, and mortality.

Statistical analysis

The relationships between scoring systems and disease severity were analyzed using the Chi-square test. A p-value of less than 0.05 was considered statistically significant. Logistic regression was used to identify variables associated with morbidity and mortality. The risk is presented as an odds ratio (OR) and a 95% confidence interval (95% CI). Variables with p<0.05 in the univariate analyses were included in multivariate analyses. SPSS Statistics version 21.0 (IBM Corp. Released 2012. IBM SPSS Statistics for Windows, Version 21.0. Armonk, NY: IBM Corp.) was used for statistical analyses. Receiver operating characteristic (ROC) curve analysis was performed by MedCal® software version 16.8.4 (MedCalc Software Ltd., Ostend, Belgium). The comparison of various scoring systems for outcome prediction was done using ROC analyses by calculating the area under the curve (AUC). An AUC of >0.5 was considered significant.

Management protocol

Fluid resuscitation with lactated Ringer's solution was done at the time of admission. Vitals and urine output were closely monitored. Early initiation of enteral feeding was done in cases of mild pancreatitis. Nasojejunal feeding was done in more severe cases when oral intake was not tolerated. Pain management was based on the WHO analgesic ladder [20].

## Results

The mean age of the patient was 39.24 ± 13.09 years, and the majority (45, 52.3%) were females. Gallstone pancreatitis was found to be the most common cause of AP (45, 52.3%), followed by alcohol-induced pancreatitis (28, 32.6%), hypertriglyceridemia (7, 8.1%), and ERCP (2, 2.3%). No etiological cause of AP could be identified in four (4.6%) patients. A significantly higher incidence of gallstone pancreatitis (p<0.001) and hypertriglyceridemia-induced pancreatitis (p=0.012) was found in females. Males had a significantly higher incidence of alcohol-induced pancreatitis (p<0.0001) (Table 2). Pain in the abdomen was found to be the most common complaint (80, 93%), followed by nausea and vomiting (39, 45.3%), abdominal distention (35, 40.6%), fever (5, 5.8%), and chest pain (2, 2.32%) at the time of presentation (Table 2). Most (38, 44.1%) patients had moderate disease severity, 26 (30.2%) had mild disease, and 22 (25.6%) had SAP. The disease severity was comparable in both genders (p=0.326). The duration of hospital stay was 9.58 ± 6.12 days. The mean duration of hospital stay of patients with severe disease was 13 ± 7.2 days, which was significantly longer than those with mild (6.81 ± 3.64 days) and moderate AP (9.50 ± 5.86 days) (p=0.001). The mortality rate of patients having SAP was significantly higher than that of patients with mild to moderately severe disease (p=0.001).

**Table 2:** Baseline characteristics of patients with AP AP: acute pancreatitis, APACHE II: Acute Physiology and Chronic Health Evaluation II, BISAP: Bedside Index of Severity in Acute Pancreatitis, mCTSI: Modified Computed Tomography Severity Index, ERCP: endoscopic retrograde cholangiopancreatography

Characteristics	Groups	Numerical value
Gender	Male	41 (47.7%)
	Female	45 (52.3%)
Age group (years)	10-20	6 (7.0%)
	20-30	22 (25.6%)
	30-40	24 (27.9%)
	40-50	19 (22.1%)
	50-60	11 (12.8%)
	60-70	4 (4.7%)
Etiology	Gallstone	45 (52.3%)
	Alcohol	28 (32.6%)
	Hypertriglyceridemia	7 (8.1%)
	Post-ERCP	2 (2.3%)
	Idiopathic	4 (4.6%)
Clinical feature	Abdominal pain	80 (93.0%)
	Nausea and vomiting	39 (45.3%)
	Abdominal distension	35 (40.6%)
	Fever	5 (5.8%)
	Chest pain	2 (2.32%)
Ranson	<3	58 (67.4%)
	≥3	28 (32.6%)
APACHE II	<8	66 (76.7%)
	≥8	20 (23.3%)
BISAP	0-3	70 (81.4%)
	4-5	16 (18.6%)
mCTSI	0-6	64 (74.4%)
	8-10	22 (25.6%)
Revised Atlanta Guidelines (2012)	Mild	26 (30.2%)
	Moderately severe	38 (44.1%)
	Severe	22 (25.6%)
Outcome	Discharged	79 (91.9%)
	Death	7 (8.1%)

**Figure 1:**
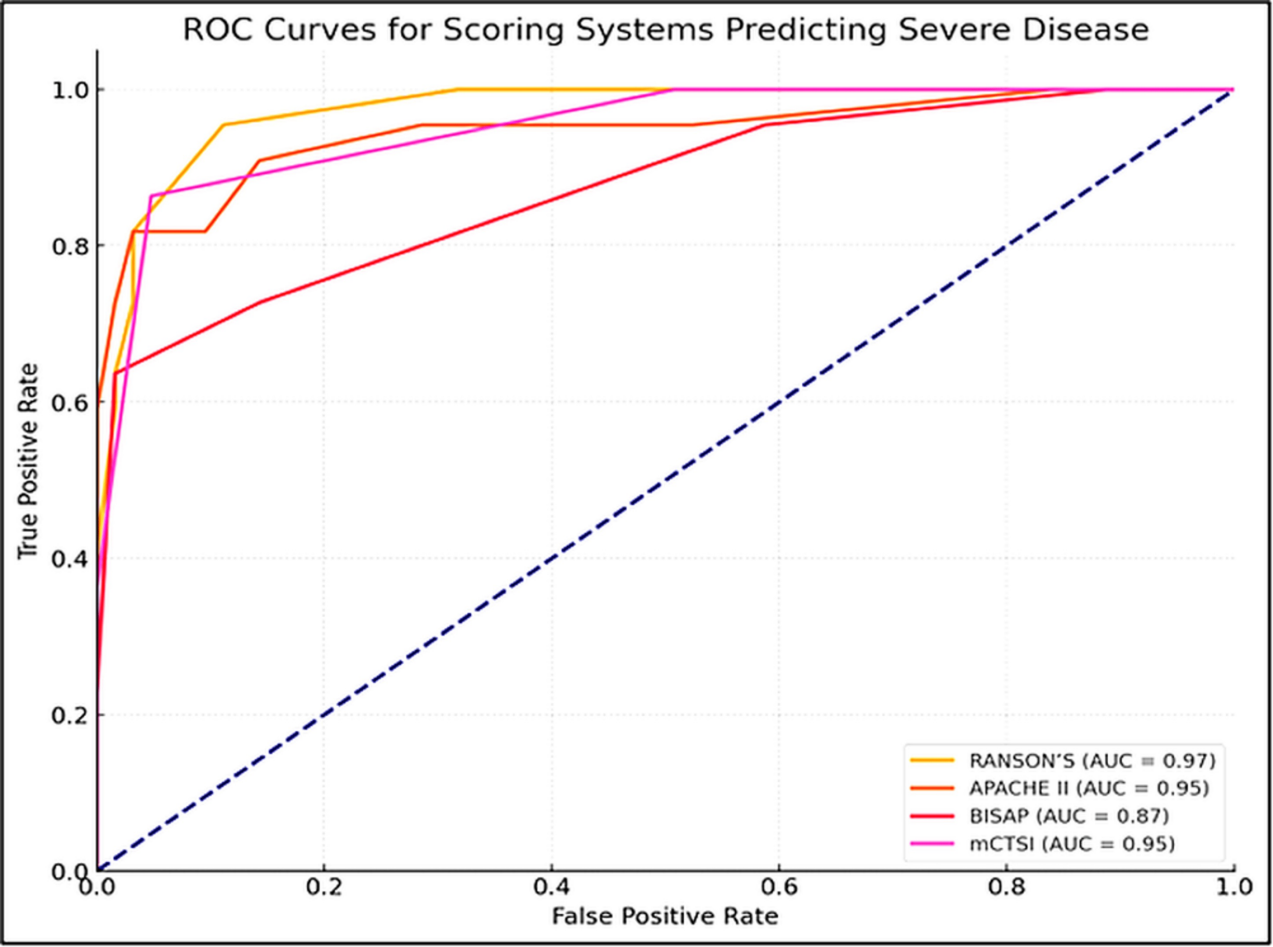
Comparison of ROC curve for predicting disease severity ROC: receiver operating characteristic, AUC: area under the curve

During the course of the disease, seven (8.1%) patients died, while 79 (91.9%) improved. The five (71.4%) patients who died had SAP, and 62 (78.5%) survivors had mild to moderate AP (Table 2). Multi-organ failure was found to be the leading cause of death in four (57.1%), and three (42.3%) of them had SAP. The mean score of all the studied scoring systems was significantly higher in patients with severe disease than those with mild to moderate attacks (p<0.001). Patients who died had significantly higher scores in all the scoring systems (Table 2). On ROC curve analysis, Ranson was found to be the best predictor of SAP (AUC: 0.97), followed by APACHE II (AUC: 0.95), mCTSI (AUC: 0.95), and BISAP (AUC: 0.87) (Figure 1, Table 3).

**Table 3:** AUC scoring system in predicting SAP, AP, and mortality AUC: area under the curve, SAP: severe acute pancreatitis, PA: acute pancreatitis, APACHE II: Acute Physiology and Chronic Health Evaluation II, BISAP: Bedside Index of Severity in Acute Pancreatitis, mCTSI: Modified Computed Tomography Severity Index

Scoring system AUC (95% CI)	Severity prediction		Pancreatic necrosis		Prediction of mortality	
Ranson	0.97 (0.94-0.99)		0.87 (0.79-0.95)		0.68 (0.54-0.80)	
APACHE II	0.95 (0.87-0.99)		0.78 (0.60-0.96)		0.72 (0.58-0.85)	
BISAP	0.87 (0.78-0.96)		0.52 (0.40-0.64)		0.67 (0.52-0.80)	
mCTSI	0.95 (0.90-0.99)		0.94 (0.88-0.99)		0.69 (0.54-0.83)	
	AUC differences	p-value	AUC differences	p-value	AUC differences	p-value
Ranson ~ APACHE II	0.02	0.78	0.09	0.02	0.04	0.64
Ranson ~ BISAP	0.10	0.01	0.35	0.01	0.01	0.92
Ranson ~ mCTSI	0.02	0.78	0.07	0.62	0.10	0.91
APACHE II ~ BISAP	0.08	0.03	0.26	0.02	0.05	0.58
APACHE II ~ mCTSI	0.00	0.99	0.16	0.02	0.03	0.73
BISAP ~ mCTSI	0.08	0.03	0.42	0.01	0.02	0.84

mCTSI was found to be the best predictor of pancreatic necrosis (AUC: 0.94), followed by Ranson (AUC: 0.87), APACHE-II (AUC: 0.78), and BISAP (AUC: 0.52). APACHE II had a slight edge over the rest of the scoring system in mortality prediction (APACHE II AUC: 0.72 95% (CI 0.58-0.85), BISAP AUC: 0.67 (CI 0.52-0.80), Ranson AUC: 0.68 (CI 0.54-0.80), and mCTSI AUC: 0.72 (CI 0.58-0.85)). Ranson score was found to have a significant advantage (p=0.01) over the BISAP on comparative analysis of the ROC curve for severity prediction. Ranson had the highest sensitivity for detecting disease severity and mortality risk; however, as expected, mCTSI had the highest sensitivity for detecting pancreatic necrosis. APACHE II had the highest specificity for detecting SAP and death, while mCTSI had the highest specificity for pancreatic necrosis. APACHE II had the highest positive predictive value for predicting SAP and mortality, while mCTSI had the highest positive predictive value for detecting pancreatic necrosis. The negative predictive value of Ranson was the highest for predicting disease severity and death. However, as expected, mCTSI had the highest score in the prediction of pancreatic necrosis (Table 4).

**Table 4:** Efficacy of different scoring systems in predicting severity and outcome of patients of AP AP: acute pancreatitis, CI: confidence interval, APACHE II: Acute Physiology and Chronic Health Evaluation II, BISAP: Bedside Index of Severity in Acute Pancreatitis, mCTSI: Modified Computed Tomography Severity Index

Predicting severity	Sensitivity (%) (95% CI)	Specificity (95% CI)	Positive predictive value (95% CI)	Negative predictive value (95% CI)
Ranson	0.95 (0.84-1.00)	0.89 (0.80-0.96)	0.75 (0.59-0.90)	0.98 (0.93-1.00)
APACHE II	0.73 (0.53-0.91)	0.98 (0.95-1.00)	0.94 (0.81-1.00)	0.91 (0.84-0.97)
BISAP	0.73 (0.53-0.91)	0.86 (0.76-0.93)	0.64 (0.43-0.81)	0.90 (0.81-0.97)
mCTSI	0.86 (0.74-0.97)	0.95 (0.89-1.00)	0.81 (0.77-0.94)	0.94 (0.90-0.99)
Outcome				
Ranson	0.79 (0.56-1.00)	0.57 (0.46-0.68)	0.76 (0.64-0.88)	0.93 (0.85-1.00)
APACHE II	0.57 (0.45-0.69)	0.88 (0.80-0.95)	0.77 (0.60-0.95)	0.91 (0.84-0.97)
BISAP	0.57 (0.31-0.82)	0.76 (0.67-0.85)	0.72 (0.55-0.89)	0.90 (0.82-0.96)
mCTSI	0.57 (0.29-0.83)	0.81 (0.71-0.89)	0.81 (0.70-0.92)	0.91 (0.82-0.97)
Pancreatic necrosis				
Ranson	0.77 (0.60-0.94)	0.86 (0.70-1.00)	0.66 (0.43-0.89)	0.88 (0.80-0.96)
APACHE II	0.58 (0.31-0.84)	0.85 (0.69-0.99)	0.69 (0.30-1.00)	0.81 (0.60-1.00)
BISAP	0.45 (0.30-0.59)	0.70 (0.51-0.89)	0.44 (0.26-0.62)	0.69 (0.50-0.88)
mCTSI	0.89 (0.81-0.97)	0.94 (0.88-1.00)	0.83 (0.69-0.97)	0.92 (0.82-1.00)

**Figure 2:**
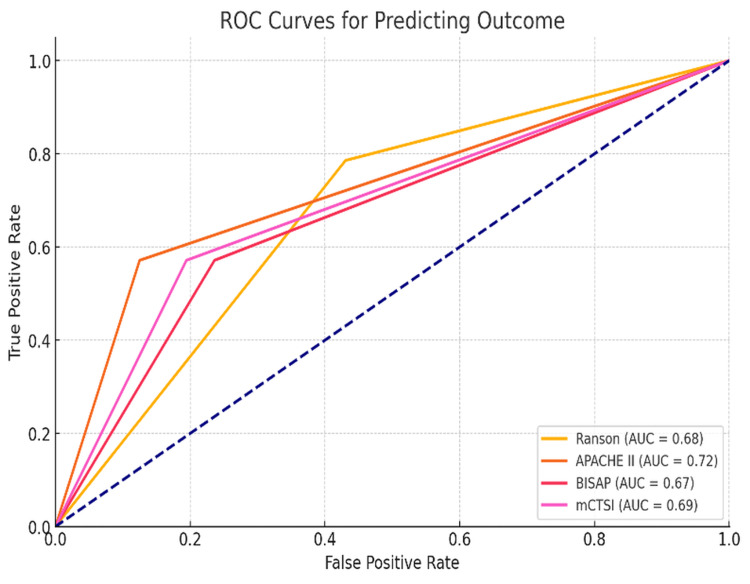
Comparison of ROC curve for predicting mortality ROC: receiver operating characteristic, AUC: area under the curve

Although there was no statistically significant difference for the prediction of SAP (p=0.99) on pairwise comparison between APACHE II and mCTSI, the latter was significantly more efficient for predicting pancreatic necrosis (AUC difference 0.16 and p=0.02). A pairwise comparison of scores revealed a higher accuracy of Ranson over BISAP (p=0.01). APACHE II was significantly better than BISAP for prognosticating SAP and pancreatic necrosis (p=0.03 and p=0.02, respectively). The accuracy of mCTSI was significantly better than BISAP for prognosticating SAP (p=0.03). As expected, mCTSI had significantly higher accuracy for predicting pancreatic necrosis (p=0.01). No statistically significant advantage of Ranson over APACHE II or mCTSI was found for predicting disease severity (p=0.78). Although APACHE II had the highest AUC for the outcome prediction on the ROC curve analysis, the differences between all the scoring systems were not statistically significant (Table 3, Figure 2).

## Discussion

This prospective study was undertaken to compare the predictive accuracy of commonly used scoring systems, i.e., APACHE II, Ranson, BISAP, and mCTSI, in predicting the severity and outcome in a cohort of patients of AP. In this study, the disease severity based on the Revised Atlanta Guidelines (2012) was mild AP in 30%, MSAP in 44%, and SAP in 26% of patients, similar to the findings of other studies [21,22]. Seven (8.1%) of the patients died, and five (71.4%) of them had severe diseases. Other studies have reported a similar case fatality rate [21,22].

Ranson in the present study had the highest AUC for predicting SAP, comparable to the observations made by Papachristou et al. [23]. Some studies have, however, reported that APACHE II had the highest AUC for severity prediction [7,21]. mCTSI was the best predictor for detecting pancreatic necrosis in our study, concordant with the observations made in some other studies [21,23,24]. We found that APACHE II had the highest AUC for mortality prediction, which agrees with some studies' observations [22,24]. However, few others have reported that the AUC of APACHE II is almost similar to Ranson [23]. In our study, when compared with other scoring systems, BISAP was found to have the lowest efficacy (AUC) in predicting disease severity, pancreatic necrosis, and outcome. Some studies, however, have reported that the accuracy of BISAP was similar to another scoring system (Ranson and APACHE II) for predicting SAP, pancreatic necrosis, and death [23,24]. Kumar et al. reported that mCTSI had the highest accuracy in predicting SAP and pancreatic necrosis [21].

In the present study, the sensitivity of Ranson was the highest for predicting SAP and death. Cho et al. also reported that Ranson has the highest sensitivity for predicting SAP compared to BISAP, APACHE II, and CTSI [7]. mCTSI had the highest sensitivity, specificity, and positive and negative predictive value for detecting pancreatic necrosis. Papachristou et al. reported that although CTSI has the highest sensitivity for predicting SAP and pancreatic necrosis, they found no statistically significant difference between Ranson, APACHE II, and CTSI for predicting death [23]. In the present study, the specificity of APACHE II was highest for predicting SAP and death. At the same time, mCTSI showed the highest specificity for pancreatic necrosis, which is concordant with the findings reported by other studies [21,23,24].

## Conclusions

AP is a serious medical condition with a high risk of morbidity and mortality. All four scoring systems, i.e., Ranson, APACHE II, BISPA, and mCTSI, have good predictive ability for both severity and mortality in patients with AP. The ROC curve analysis demonstrated that Ranson was superior to the other scoring systems for predicting severity, and APACHE II had the highest accuracy for mortality. Although Ranson and APACHE II have a definite advantage over all the scoring systems, BISAP might be preferred over Ranson and APACHE II in clinical practice due to its ease of use, as it is less complex. Despite its limitations, the mCTSI score might be preferred in clinical practice for predicting disease severity due to its wide availability and feasibility.
